# The impact of Covid-19 on the well-being of young people with conduct disorder and their families

**DOI:** 10.1192/j.eurpsy.2022.858

**Published:** 2022-09-01

**Authors:** M. Bull, M. Livanou, R. Ball

**Affiliations:** Kingston University, Psychology, London, United Kingdom

**Keywords:** Conduct Disorder, Conduct problems, Behavioural disorders, Covid-19

## Abstract

**Introduction:**

The Covid-19 pandemic has significantly changed family dynamics and parents experience greater psychological distress. Conduct problems in young people have increased by 35%. However, it is not known how Covid-19 associated stresses have affected parenting practice, conduct problems, and comorbidities and what additional support is needed for families at risk.

**Objectives:**

This study uses self-report measures and semi-structured interviews to examine and explore the impact of Covid-19 on the families of young people with conduct problems and comorbid mental health conditions.

**Methods:**

This is a sequential mixed-methods study. Eligible families with children aged between 11-18 years have participated. One-hundred-and-eighty-two families have completed eight online questionnaires and 12 have participated in semi-structured follow-up interviews.

**Results:**

Analyses indicate that parental harshness, warmth, educational background, and employment have a significant impact on Covid-19 exposure and worries, as well as significantly higher scores of conduct disorder symptoms. Interview codes reveal that young peoples’ behaviour became more severe during the pandemic, and this was associated with reduced in-person support services, reduced personal space at home, and parents taking on the additional role of educator.

**Conclusions:**

The findings suggest that Covid-19 is a significant risk factor to young people with conduct problems and their families. For example, reduced parental warmth and increased parental harshness increased conduct problems for young people during the lockdown. This study highlights that policies and services should work to better support such families. Future online psychosocial interventions are needed to empower families and improve parenting practice at home during the lockdown period and in general.

**Disclosure:**

No significant relationships.

